# Editorial: Standard employment enclaves, precarity and informality: explaining employment configurations in the global south

**DOI:** 10.3389/fsoc.2026.1822329

**Published:** 2026-04-01

**Authors:** Irene Dingeldey, Heiner Fechner, Dasten Julián-Vejar

**Affiliations:** 1 Institute of Labour and Economy, University of Bremen, Bremen, Germany; 2 Chamber of Employees in Bremen, Bremen, Germany; 3 Centro de Investigaciones y Estudios Avanzados del Maule, Universidad Católica del Maule, Talca, Chile

**Keywords:** employment, global south, informal work, power resource approach, regional studies, regulation, segmentation

The discourse on labor market segmentation has yielded many insights, especially regarding the vulnerability of children, women, ethnic minorities, Indigenous people and migrants. These groups are often exposed to exhausting working conditions and non-subsistent wages. However, much of this debate is oriented toward developments in the Global North, while research on the Global South has largely focused on the dualisation of formal and informal work or on single forms of employment such as contract work. As a result, we still know little about specific patterns of segmentation in the Global South, the full spectrum of employment forms and transitions between segments, and the ways segmentation manifests in particular industries and regions, both within and beyond formal employment. This includes the role of national labor law and international labor standards, such as those of the International Labor Organization (ILO), and the challenges and success of unions and other collective actors in organizing workers amid blurred boundaries between formal employment, self-employment and informal work. To address these questions and enrich the segmentation debate with a Global South perspective, we launched this Research Topic.

The contributions cover case studies on Latin American African and Asian countries as well as analyses of the automotive, textile, financial and transport sector. They address forced domestic labor, working-class actions and interests, the effects and challenges of COVID-19 for labor markets, occupational health, the spread of digital platform work, and the links between work and territory. Together, these approaches to precariousness, informality, and segmentation provide a broad set of conceptual and methodological tools to understand transformations of work in its global dimension and to highlight major challenges for labor regulation. The contributions call for legislation, policies and programmes that counter the erosion of rights to decent and dignified work and use the visibility of evolving segmentation trends to develop solutions that ensure quality employment through oversight, dialogue and worker action.

Martínez-Martínez et al. analyse changes in labor formality rates before and 2 years after the main COVID-19 contingency measures in nine Latin American countries. They find that the weighted labor formality rate increased, which they attribute to greater capital accumulation, integration of productive, and commercialization processes and differentiated fiscal incentives. At the same time, they underscore the precarious situation of women. They point to the need for public policies that reverse current labor market conditions and strengthen resilience to future shocks, with special emphasis on the informal sector and women.

A sectoral analysis by Dingeldey and Schäfer examines formal employment, mainly permanent full-time work as a standard employment relationship, in South Africa's automotive industry. Applying a power resource approach and using secondary data and expert interviews, they show that labor standards and pay levels vary along the regional value chain, reflecting differences in trade union power that mirror structural power differences between OEMs and suppliers.

Employment conditions in a South African financial services institution appear more casual than in the automotive sector and are accompanied by high turnover. Vilakazi et al. use a qualitative case study to explore how decent work affects retention, identifying four perceptions of decent work: security-based, relationship-centered, compensation and recognition-focused, and development and growth-oriented. Accordingly, career development and growth opportunities, recognition and reward systems, work-life balance and flexible arrangements, compensation and benefits, job security and stability, and alignment of company culture and values, are suggested to inform comprehensive retention strategies based on promoting decent work practices.

Jörg Nowak analyses the situation of self-employed truckers in the Brazilian road transport, a sector with a history of mobilization within reactionary and conservative movements in Latin America. Engaging with theories of working-class power that presuppose clear worker interests, he argues that such interests emerge through political processes and examines the Brazilian context of self-employed truckers and taxi drivers who see themselves as their own bosses. Although they share interests with a smaller group of formally employed truckers, these self-employed workers often mobilize together with employers and transport companies, making their alliances and mobilization patterns expressions of ambiguous and dual class interests. The article thus sheds light on conditions of organization and representation in a context where the working class cannot be treated as homogeneous.

Stecher et al. reconstruct the labor market of delivery and ride-hailing digital labor platforms using data collected between 2019 and 2025, including worker and key-informant interviews, documentary analysis, secondary sources and field observations. They show how platform companies operate in Latin American contexts, the regulatory challenges they pose, and the global debates on minimum standards for precarious platform work. The authors identify three central characteristics of this labor market that concentrate its tensions, contradictions and specificities in the Global South, and highlight workers' ambivalent position between autonomy and demands for rights in a culture oriented toward dependent employment.

Schalkowski explores political campaigns in Peru aimed at legislative reforms against forced labor, linked to the consolidation of organized domestic workers. She analyses how international labor norms on forced labor are disseminated, appropriated and politically mobilized at the national level, and how this process shapes labor regulation, policy responses and trade union action. Focusing on interactions between the ILO and national domestic workers' unions, she indicates how international law and agreements are translated and negotiated locally, creating spaces for advocacy, mobilization and negotiation for a feminized and racialised sector. The study illustrates the intersection of “universal” labor standards, knowledge production and trade union mobilization in this field.

Khan et al. use a cross-sectional survey of 541 participants and the Respiratory Health Questionnaire to study women workers in a highly toxic and polluting textile industry. They document pronounced health risks and highlight the need for policy interventions to improve working conditions, enforce minimum wage and hours standards, and provide social security benefits for this vulnerable worker group. Given the industry's role in global export chains, their findings offer a crucial reference point for due diligence and corporate responsibility initiatives regarding human rights at work.

Idrus et al. apply a sustainable livelihood approach with a gender-social inclusion perspective to child labor prevention and remediation in rural South Sulawesi, Indonesia. Using qualitative methods and Participatory Rural Appraisal techniques in three regions with different topographies, they show that child labor is driven by poverty, household characteristics, parents' education and employment, and limited access to education and labor markets. They identify social, natural, financial and human capitals as key resources for prevention and management, and propose rethinking predominantly regulatory and punitive, centralized models that often prove ineffective. Instead, they argue for involving communities and volunteer groups more directly in monitoring and remediation, opening a new paradigm for local appropriation of international conventions in a context where forms of child labor may be becoming more brutal and consequential.

With a territorial focus, Lima et al. compare two empirical cases in the Brazilian clothing sector using interviews with owners and workers. They show how informal production hubs are reinterpreted through a model of individual self-entrepreneurship aimed at reducing costs and increasing national competitiveness, and argue that the state shapes work territories via regulation, promotion or exclusion through various policies. In territories marked by longstanding precariousness, such state intervention is perceived as a positive development.

Finally, Blanco et al. examine regional labor markets in the Chilean regions of Antofagasta and La Araucanía, emphasizing both structural similarities and differences. Using multivariate sociodemographic, contractual and occupational data, they identify region-specific and common elements, arguing that Chile's geographic and productive diversity requires a territorial analysis of segmentation processes. They highlight the strategic importance of extractive sectors such as mining and forestry for understanding regional work cultures, occupational structures and labor segmentation, especially regarding racialisation of Indigenous people and migrants, gender inequalities and urban–rural gaps, and call for territorially differentiated policies that account for structural diversity in employment conditions.

